# Identification, classification, and characterization of alpha and beta subunits of LVP1 protein from the venom gland of four Iranian scorpion species

**DOI:** 10.1038/s41598-023-49556-6

**Published:** 2023-12-14

**Authors:** Fatemeh Salabi, Babak Vazirianzadeh, Masoumeh Baradaran

**Affiliations:** 1 Razi Vaccine and Serum Research Institute, Agricultural Research, Education and Extension Organization (AREEO), Ahvaz, Iran; 2 Social Determinant of Health Research Center, Ahvaz Jundishapur University of Medical Sciences, Ahvaz, Iran; 3 Toxicology Research Center, Medical Basic Sciences Research Institute, Ahvaz Jundishapur University of Medical Sciences, Ahvaz, Iran

**Keywords:** Computational biology and bioinformatics, Biological techniques, Biotechnology, Animal biotechnology, Sequencing

## Abstract

Scorpion venoms contain bioactive peptides and proteins. Some, can be used for pharmaceutical purposes. So, identification of venom proteins matters because, in addition to determining the function of the toxins can also be an excellent guide to developing new drugs. Here, we got transcriptome of venom glands from four Iranian scorpion species, including *Hemsicorpius lepturus*, *Mesobuthus eupeus*, *Andructunus crassicuada*, and *Hottentotta saulcyi* using cDNA library synthesis and high-throughput transcriptomic analysis of the venom glands. In a comparative way, we identified the cDNA encoding isoforms of subunits (alpha and beta) of BotLVP1/BmLVP1-like protein in the venom gland of three species except for *H. lepturus*. Characterization and structure determination of the LVP1_like proteins combined with gene map analysis provided evidence of the existence of some isoforms of LVP1_like proteins, encoded by genes with two exons and one intron, which can be classified in CSαβ superfamily in the venom gland of three Iranian scorpion species. According to the high similarity with BotLVP1 and BmLVP1, these proteins could also be potent to mediate cholesterol homeostasis. However, further research is needed to prove it, and this study just may lay the foundation lead to light up this way.

## Introduction

Scorpions and scorpion stings have taken an essential part in public health all over the world, including in Iran. The frequency of scorpion stings is very significant in Iran, especially in the southern and southwestern regions of the country^1^. Most cases of scorpion stings have been reported from six species of scorpions, including *Mesobuthus eupeus*, *Hemiscorpius lepturus*, *Hottentotta saulcyi*, *Odonthobuthus doriae*, *Androctonus crassicauda* and *Hottentotta schach*^2^. Therefore, the antivenom produced by the Razi Institute of Vaccine and Serum Production in Karaj, Iran, for the treating scorpion stings is prepared using a mixture of venom extracted from these six medically important species^3^.

Epithelial cells of the scorpion's venom gland synthesize components of the venom and release them into the lumen of the gland where stores them^4^. These compounds comprise peptides, proteins, enzymes, amines, and nucleotides^5^. Scorpions use these compounds for defense, feeding, reproduction, and overall survival^6^. Nevertheless, recent research on determining the function and potential of these compounds has shown that although some neurotoxins and cytotoxins in the venom gland of scorpions threaten human life, many venom peptides and proteins revealed a broad-spectrum bioactivity^7,8^ in the treatment of many diseases including, cancers^9,10^, infections^11^, and cardiovascular disorders^12^, epilepsy^13^, autoimmune and inflammatory disease^14^.

There is also some evidence about the presence of the compounds interfering with lipid metabolism in the venom gland of scorpions. An increase in serum free fatty acids and phospholipids; and a reduction in total cholesterol in canine injected by scorpion venom^15^, inhibiting the activity of HMG-CoA reductase by a protein, Lipolysis-activating peptide 1-beta (BmLVP1_beta,or bumarsin), isolated from *Mesobuthus martensii* venom gland^16^, and the presence of several Lipolysis activating peptides(LVP1s) in venom glands of some scorpion including Chinese *Buthus martensii*^17^, *Lychas mucronatus*^18^, and *Buthus occitanus tunetanus*^19^ have been proved.

LVP1 is a lipolytic peptide that was first isolated from the venom of *Buthus occitanus* and named BotLVP1. Some experiments on BotLVP1, revealed that it is a heterodimeric protein with alpha and beta subunits structurally related to Na^+^ channel toxins but is not toxic and can stimulate lipolysis in rat adipocytes^20,21^.

Further research identified two cDNA encoding subunits α and β of a BotLVP1-like peptide (BmLVP1) from the venom gland of *M. martensii*. The heterodimer structure determined for BmLVP1 further supported the relationship between BmLVP1 subunits and BotLVP1 subunits^22^.

More research indicated that two LVP1-beta subunits originated from *M. martensii* form a homodimer named bumarcin, which significantly inhibited HMG-CoA reductase compared to simvastatin^16^.

All in all, LVP1 was considered a candidate for the production of an alternative drug for the treatment of hyperlipidemia^16^. According to the importance of this protein, in this study, the venom gland transcriptome of four medically important scorpion species, including *H. saulcyi*, *M. eupeus*, *A. crassicauda*, and *H. lepturus* was analyzed to find possible isoforms of two subunits (alpha and beta) of LVP1. In addition, the characteristics of LVP1 subunits isoforms and their three-dimensional structures, were determined in different scorpion species. Furthermore, the exon–intron map of the gene encoding this protein was also determined.

## Materials and methods

### Sample preparation

Whatever will be explained here about *H. saulcyi* has previously been done for *H. lepturus*, *A. crassicauda*^23^, and *M. eupeus*^24^ using the same methodology.

Scorpions of species *H. saulcyi* were collected from the deserts around Khuzestan province, southwest of Iran, and were transferred to the laboratory of the Toxicology research center located in Ahvaz Jundishapur University of Medical Sciences for identification and confirmation of the species according to the morphological properties^25^. Confirmed scorpion samples were milked by electrical shock, 72 h later, they were transferred to the molecular laboratory for RNA extraction.

### RNA extraction and cDNA library synthesis

A total of twenty specimens of *H. saulcyi* were captured in summer 2022. Considering that based on the general guidelines for RNA-Seq, at least three biological replicates are required to generate a reliable RNA-Seq analysis^26^, we selected six mature scorpions out of all captured specimens to construct cDNA libraries. The terminal segment (telson) of selected scorpions was separated and powdered with the help of the liquid nitrogen. Total RNA was extracted under sterile conditions using RNeasy Mini Kit (Cat. 74104) according to the manufacturer’s instructions. The quality of extracted RNAs were evaluated using Nanodrop (co. Thermo, USA). Each of the three RNA samples were pooled in equal concentrations to form two samples prior to sequencing^27^. The RNA Integrity Number (RIN) of two pooled RNA samples was determined by Macrogen, Inc. (Seoul, Korea) using the Agilent 2100 Bioanalyzer System (Agilent Technologies, USA) according to the manufacturer's instructions. Two pooled RNA samples with appropriate RIN (A RIN value higher than 7) were used to prepare rRNA-depleted libraries using the TruSeq® Stranded mRNA Library Prep (cat. number 20020594) according to the manufacturer's instructions. Finally, the Illumina HiSeq 2000 platform was used to sequence cDNA libraries with 150 bp paired-end reads.

### Transcriptome assembly and annotation

First, the quality of the raw RNA-Seq reads was assessed with FastQC v0.11.5 (http://www.bioinformatics.bbsrc.ac.uk/projects/fastqc) using default parameters. Subsequently, reads were subjected to TRIMMOMATIC v0.36 to remove low-quality reads and any Illumina adapter remnants, with the following settings: ILLUMINACLIP:TruSeq3-PE.fa:2:30:10 LEADING:25 TRAILING:25 MAXINFO:90:0.90 AVGQUAL:20 MINLEN:50. Finally, the FastQC was performed to verify the final quality of the dataset.

A de novo assembly of clean reads was then performed using Trinity software (v2.10.0)^28,29^ with a minimum contig length of 200 bp. Since no complementary genome of the studied scorpion species and no reference genome from a closely related species is available, the de novo transcriptome assembly method was used to determine the transcripts. The reads were mapped to transcriptome assemblies using Bowtie2 v2.3.5.1, under default parameters. Finally, TransDecoder v5.5.0 (https://github.com/TransDecoder/TransDecoder/releases) was employed to predict candidate coding regions in transcript sequences, discarding possible non-coding RNA and DNA contamination with “-t″ option for Transdecoder.LongOrfs and Transdecoder.Predict^29^.

### Characterization of LVP1 subunits in scorpion species

In order to identify the proteins and nucleotides with the greatest sequence similarity to protein LVP1, alpha and beta subunits originated from scorpions and closely related species were manually searched. With Blastx and Blastp and the E value threshold of le−3, the TransDecor-predicted proteins and nucleotide sequences were searched against the local LVP1 alpha and beta database. Finally, the homologous sequences related to LVP1 alpha and beta were selected from mRNA candidates of all four scorpion species. These sequences were classified based on similarity to already known families/classes. For this purpose, newly extracted sequences were searched against NCBI and UniProt databases. A sequence alignment was done using the EBI online server (https://www.ebi.ac.uk/Tools/msa/clustalo/). Amino acid alignment of isoforms was done using BioEdit software (https://www.bioedit.com/).

### Characterization of the gene and protein of LVP1-alpha and LVP1-beta

Characterization of the gene and protein of the predicted LVP1-alpha and LVP1-beta proteins not only increases the confidence in the sequences but also provides more complete information about the obtained proteins.

An intron–exon map of LVP1-alpha and LVP1-beta were predicted by comparing whole genome shotgun sequences with mRNAs, as described previously^30^. The mRNA sequence of KTXLP2 (alternative name: LVP1-beta) (AF155368.1) with its genomic DNA from contig339532 of whole genome shotgun sequence (AYEL01080250.1) from *Mesobuthus martensii* along with similar sequence originated from *Hottentota trilineatus* (SRX815889) were used as templates to compare with the LVP1-alpha and LVP1-beta mRNA sequences found in the current study. A sequence alignment was created with MAFT (high-speed multiple sequence alignment program) online server in EBI (https://mafft.cbrc.jp/alignment/server/index.html). In the following, potent signal peptides of all predicted HMG-CoA reductase inhibitors were predicted by SignalP-6.0 available on server https://services.healthtech.dtu.dk/service.php?SignalP. Physicochemical properties of mature proteins of LVP1-alpha and LVP1-beta, including molecular weight, Iso-electric point, half-life, water solubility, and net charge, were determined with the Protparam tool from Expasy (https://web.expasy.org/protparam/) and proteomics tool from INNOVAGEN (http://pepcalc.com). Disulfide bridge patterns were determined using the DISULFIND server (https://bio.tools/disulfind).

### Determination the 3D-structure of LVP1-alpha and LVP1-beta

The 3D-structure of the newly identified LVP1-alpha and LVP1-beta isoforms was determined via Homology modeling. For each protein, four models were generated using four different online modeling servers as follows: I-TASSER by multiple threading approaches, PHYRE2 with Poing^2^ method, Robetta using RoseTTAFold, and SWISS-MODEL. Quality assessment of obtained structures was employed using the overall quality factor on SAVESv6.0 (https://saves.mbi.ucla.edu/) as well as by Z-score through the ProSA-web (https://prosa.services.came.sbg.ac.at/prosa.php) to select the best model for each protein. A more negative Z-scores represents a more validated structure^31^. The selected protein structures were energetically minimized using the YASARA Minimization Server (http://www.yasara.org/minimizationserver.htm). Finally, to determine whether the predicted protein structures have the permitted torsional angles, the Ramachandran plot was drawn using the PSVS server (https://montelionelab.chem.rpi.edu/PSVS/PSVS/).

### Phylogenetic analysis

By searching newly identified LVP1-alpha and LVP1-beta proteins against the NCBI database using BlastP, homologous peptides of these proteins were extracted. Subsequently, a multispecies sequence alignment of LVP1-alpha and LVP1-beta amino acid sequences was created using the MUSCLE tool at Mega11 software^32^. LVP1-alpha and LVP1-beta originated from different scorpion species were used in this alignment. Ultimately, this multiple sequence alignment was utilized to generate a phylogenetic tree by the neighbor-joining algorithm^32^. The neighbor-joining tree was constructed under the Jones-Taylor-Thornton (JTT) model with 1000 bootstrap replicates.

### Ethics approval and consent to participate

Permission was obtained from the Environmental Protection Agency of Iran to collect scorpions of *A. crassicauda*, *H. saulcyi*, and *H. lepturus*. No animals were euthanized as part of this study, and all sample collection methods and experimental procedures described herein were rigorously reviewed and approved by the Ahvaz Jundishapur University of Medical Sciences (Ethical code: IR.AJUMS.REC.1400.557 and IR.AJUMS.REC.1398.785), the Institutional Animal Care Committee of Razi Vaccine and Serum Research Institute (Permit number IR.RVSRI.REC.1401.017) and AREEO protocols, which comply with Iran guidelines for work with animals. This study also adheres to the ARRIVE Guidelines for reporting animal research.

## Results

### Assembly and annotation of raw sequences obtained from RNA sequencing

The transcriptome of *H. saulcyi* obtained from RNA sequencing in this study was analyzed along with the transcriptome of three additional scorpions, *H. lepturus*, *A. crassicauda*^30^, and *M. eupeus*^24^, obtained using precisely the same methodology in the previous studies.

Through Illumina paired-end sequencing of venom glands of *H. saulcyi*, 97 million paired 150 bp clean reads were obtained after adapter and low-quality reads trimming. Using Trinity de novo assembler 191,150 contigs greater than 200 bp in length were produced, including different isoforms per contig. The contigs were assembled into 110,126 unigenes. Finally, we identified 98,365 potential coding sequences in venom glands of *H. saulcyi* transcriptome assemblies. Most of these protein-coding sequences (13,723) were matched to non-redundant proteins (Nrs), Swissprot, and Pfam databases. This value was 11,415 and 11,869 transcripts for *A. crassicauda* and *H. lepturus*, respectively^23^.

### Identification of the LVP1-alpha and LVP1-beta proteins

A local database of LVP1-alpha and LVP1-beta proteins was generated by collecting the known sequences of LVP1-alpha and LVP1-beta from different scorpion species (Table 1).

**Table 1:** Lipolysis activating peptide sequences used to generate the multiple sequence alignment for detection the probable similar peptides based on the conserved sequences and phylogenetic analysis.

Taxon	Protein name	GenBank ID
*Buthus occitanus tunetanus*	Lipolysis activating peptide 1-alpha chain(BotLVP1-alpha)	P84809
*Mesobuthus martensii*	Lipolysis activating peptide 1-alpha chain(BmLVP1-alpha)	Q6WJF5
*Buthus occitanus israelis*	Lipolysis activating peptide 1-alpha chain(BoiLVP1-alpha) or Putative excitatory toxin Tx135 (BoiTx135)	B8XH01
*Lychas mucronatus*	Lipolysis activating peptide 1-alpha chain (LVP1-alpha or Neurotoxin LmNaTx7)	D9U299
*Buthus occitanus tunetanus*	Lipolysis activating peptide 1-beta chain (BoitLVP1-beta)	P84810
*Mesobuthus martensii*	Lipolysis activating peptide 1-beta chain (HMG-CoA reductase inhibitor or bumarsin)	Q95P90
*Buthus occitanus israelis*	Lipolysis activating peptide 1-beta chain (BoiLVP1-beta or beta-like toxin Tx457)	B8XGZ8
*Lychas mucronatus*	Lipolysis activating peptide 1-beta chain (LVP1-beta or Neurotoxin or LmNaTx19)	D9U2A2

Using our local LVP1 database, we conducted exhaustive BLAST searches of the *H. saulcyi*, *H. lepturus*, *M.eupeus*, and *A. crassicauda* venom glands transcriptome and found some transcripts with high sequence similarity to LVP1-alpha and LVP1-beta. In order to increase the confidence of sequence identification and to classify the sequences, the obtained sequences were directly searched against Uniprot and NCBI databases. All sequences with high sequence identity with any previously classified LVP1-alpha and LVP1-beta sequences belonging to any recognized species were considered members of this group. Accordingly, we found that scorpions encode some isoforms of alpha and beta subunits of LVP1. The cDNA and protein sequences of all isoforms of LVP1-alpha and LVP1-beta reported here have been deposited in the GeneBank database under specific names and Accession numbers (Table 2). The homologous sequence of alpha and beta subunits of LVP1 protein was not found in the venom gland transcriptome of *H. lepturus*. In contrast, in transcriptomes of *H. saulcyi*, *M. eupeus*, and *A. crassicauda* we detected a total of three isoforms of LVP1-beta subunits (OP612332, OP612333, and OP612334), two isoforms of alpha and beta subunits of LVP1 (KU513844 and KU513847), and five isoforms of alpha and beta subunits (two alpha and three beta; OP609899, OP609900, OP609901, OP609902, OP609903), respectively.

**Table 2:** Identified and submitted isoforms of LVP1-alpha and LVP1-beta in GeneBank database.

Scorpion species	Protein name	Accession number
*A. crassicauda*	LVP1-alpha_isoform1(AcLVP1-alpha_isoform1 or AcLVP1_alph1)	OP609899
*A. crassicauda*	LVP1-alpha_isoform2(AcLVP1-alpha_isoform2 or AcLVP1_alph2)	OP609900
*M. eupeus*	LVP1-alpha_isoform1(MeLVP1-alpha_isoform1 or MeLVP1_alpha1 or meuPep27)	KU513844
*A. crassicauda*	LVP1-beta_isoform1(AcLVP1-beta_isoform1 or AcLVP1_beta1)	OP609901
*A. crassicauda*	LVP1-beta_isoform2(AcLVP1-beta_isoform2 or AcLVP1_beta2)	OP609902
*A. crassicauda*	LVP1-beta_isoform3(AcLVP1-beta_isoform3 or AcLVP1_beta3)	OP609903
*M. eupeus*	LVP1-beta_isoform1(MeLVP1-beta_isoform1 or MeLVP1_beta1 or meuPep28)	KU513847
*H. saulcyi*	LVP1-beta_isoform1(HsLVP1-beta_isoform1 or HsLVP1_beta1)	OP612332
*H. saulcyi*	LVP1-beta_isoform2(HsLVP1-beta_isoform2 or HsLVP1_beta2)	OP612333
*H. saulcyi*	LVP1-beta_isoform3(HsLVP1-beta_isoform3 or HsLVP1_beta3)	OP612334
*H. lepturus*	–	–

All identified proteins showed sequence similarities to the known alpha or beta-type of lipolysis activating peptide (LVP1) from other scorpions. BmLVP1-alpha and BmLVP1-beta from scorpion *Mesobuthus martensii* and BotLVP1-alpha and BotLVP1-beta from *Buthus occitanus* are well-studied scorpion LVP1s. Accordingly, the similarity of the identified proteins with these proteins was used as the criterion and is reported in Table 3.

**Table 3:** Identity of here identified proteins with well-known LVP1s subunit alpha and beta.

Protein name	Identity with BmLVP1-alpha (%)	Identity with BotLVP1-alpha (%)	Identity with BmLVP1-beta (%)	Identity with BotLVP1-beta (%)
AcLVP1_alph1	63.04	70.65	–	–
AcLVP1_alph2	65.22	70.65	–	–
MeLVP1_alpha1	88.66	72.16	–	–
AcLVP1_beta1	–	–	67.47	59.52
AcLVP1_beta2	–	–	50	53.93
AcLVP1_beta3	–	–	58.51	58.06
HsLVP1_beta1	–	–	58.43	51.65
HsLVP1_beta2	–	–	55.43	56.52
HsLVP1_beta3	–	–	65.22	59.78
MeLVP1_beta1	–	–	75.53	61.05

**Figure 1:**
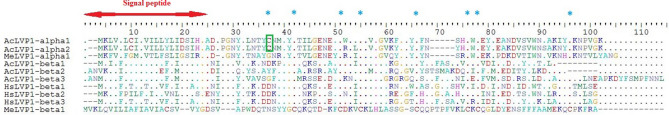
Amino acid alignment of identified LVP1-alphas and LVP1-betas from three scorpion species: *H. saulcyi*, *A. crassicauda*, and *M. eupeus.* Signal peptides are indicated with a red straight line leading to an arrow. Dots and dashes represent identical and deleted amino acids, respectively. Cysteine residues are indicated by blue asterisks. AcLVP1_alpha1 and AcLVP1_alpha2 consist of eight cysteine residues, while the other proteins consist of six cysteine residues. The green rectangle highlights two more cysteine residues in AcLVP1_alpha1 and AcLVP1_alpha2.

Amino acids alignment of identified LVP1-alphas and LVP1-betas are shown in Fig. 1. This alignment clarified that except for AcLVP1_alph1 and AcLVP1_alph2, which consist of eight cysteine residues, all other proteins contain six cysteine residues (Fig. 1).

### Exon–intron map of scorpion LVP1s

**Figure 2:**
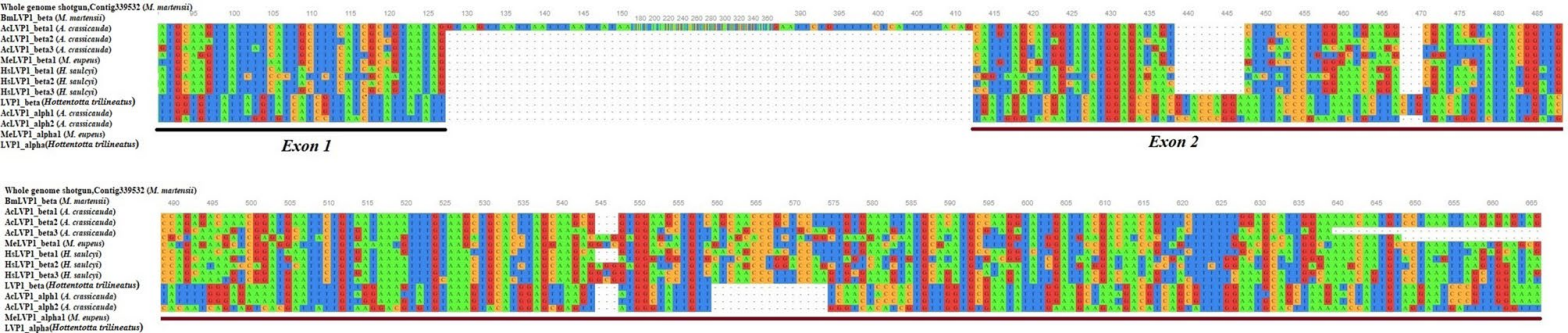
Exon–intron map of LVP1 alpha and beta gene. Accession numbers from top to bottom: AYEL01080250.1, AF155368.1, OP609901, OP609902, OP609903, KU513847, OP612332, OP612333, OP612334, SRX815889, OP609899, OP609900, KU513844, SRX815889. Coloured box parts are nucleotides of exons and the space between two exons is the only intron of these genes.

In order to organization the exon–intron patterns of LVP1 subunits alpha and beta genes (Fig. 2), the mRNA sequences of LVP1 beta or alpha discovered in this study and previously identified similar sequences originated from *Hottentota trilineatus* were aligned with the genomic and mRNA sequences of LVP1 proteins from *M. martensii* using MAFT program. The total nucleotide sequence of both LVP1-alpha and LVP1-beta from START-codon (ATG) to STOP-codon (TAA) is around 665 bp (Fig. 2). The LVP1 gene was found to consist of 2 coding exons and one intron. Exon 1 runs from nucleotide 91 to 127 and consists of 36 nucleotides, while the more extended exon 2 spans from nucleotide 413 to 665 and comprises 253 nucleotides. The exons and intron of LVP1 genes follow the standard form of GT-AG.

### Tertiary structure and physicochemical properties of LVP1-alpha and LVP1-beta isoforms

By identifying and removing the signal peptide from the sequence of identified LVP1-alpha and LVP1-beta proteins, mature proteins were obtained. Then, the determination of physical and chemical characteristics, as well as the determination of the three-dimensional structure of mature proteins, were carried out.

Physicochemical properties, including molecular weight, theoretical pI, half-life in mammalian reticulocytes, water solubility, and instability index of all found proteins, are described in Table 4.

**Table 4:** Physicochemical properties of identified alpha and beta subunits of LVP1.

Scorpion species	Scorpion species	Mature peptide (aa)	Molecular weight (g/mol)	Theoretical pI	half-life in mammalian reticulocytes (h)	Water solubility	instability index
*A. crassicauda*	AcLVP1_alph1	72 aa	8316.53	6.89	4.4	Poor	23.52 (stable)
	AcLVP1_alph2	72aa	8423.65	8.16	4.4	Poor	21.95 (stable)
	AcLVP1_beta1	73aa	8511.55	8.20	1.1	Good	8.31 (stable)
	AcLVP1_beta2	64aa	7288.30	6.71	1.1	Good	28.78 (stable)
	AcLVP1_beta3	87aa	9894.32	6.72	1.1	Poor	72.26
*M. eupeus*	MeLVP1_alpha1	67aa	7819.94	6.72	1.1	Good	59.67 (unstable)
	MeLVP1_beta1	87aa	9894.37	6.72	1.1	Poor	72.26 (unstable)
*H. saulcyi*	HsLVP1_beta1	72aa	8198.33	8.22	1.1	Good	28.88 (stable)
	HsLVP1_beta2	72aa	8269.26	4.56	1.1	Good	48.35 (unstable)
	HsLVP1_beta3	73aa	8371.45	8.24	1	Good	46.50(unstable)

**Figure 3:**
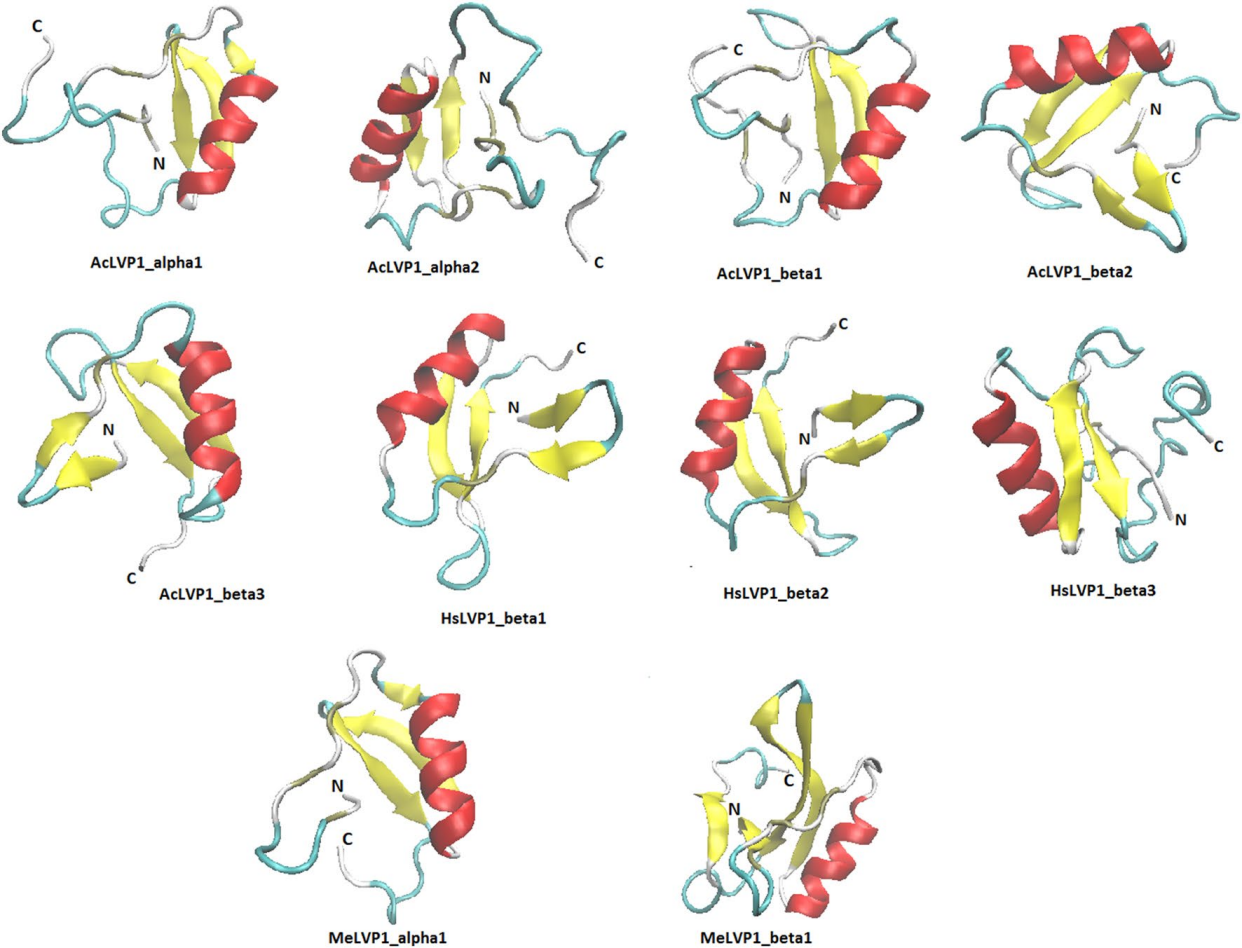
Three-dimensional structure of LVP1-alphas and LVP1-betas originated from *A. crassicauda*, *H. saulcyi*, and *M. eupeus* venom gland.

The tertiary structure of all identified LVP1-alpha and LVP1-beta proteins were predicted and illustrated in Fig. 3. For each protein, the structure with the best quality point and the most negative score among the structures made by four servers was selected as the structure of that protein. What Fig. 3 shows is the selected protein structures after energy minimization.

As seen in this figure, the LCN-type cysteine-stabilized alpha/beta (CS-α/β) domain is a common domain seen in all LVP1-alpha and LVP1-beta proteins. This domain consists of one or two alpha helixes connected to a two or triple-stranded beta-sheet through three or four disulfide bonds^33^.

What is clear is that, except for MeLVP1_alpha1 and AcLVP1_alpha1 proteins, whose beta-sheet of CS-α/β domain is three-stranded, the rest of the identified proteins have a double-stranded beta-sheet.

As mentioned above, AcLVP1_alph1 and AcLVP1_alph2 consist of 8 cysteine residues, and the other LVP1-alpha and LVP1-beta isoforms contain six cysteine residues, which indicate the formation of 3 or 4 disulfide bonds in the tertiary structure of these proteins, respectively.

**Figure 4:**
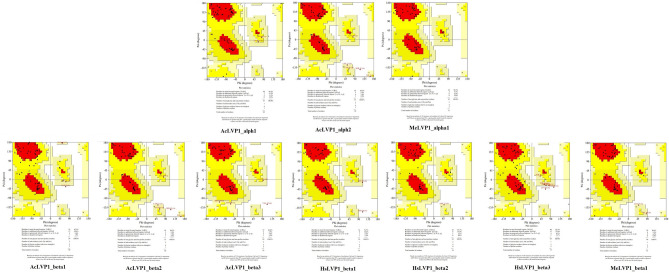
Ramachandran plot of predicted protein structures. LVP1-alphas are shown in above and LVP1-betas in bottom.

Ramachandran plot analysis (Fig. 4) revealed that more than 97% of all amino acids of predicted protein structures are located in the allowed regions, which verify the quality of the predicted models.

**Figure 5:**
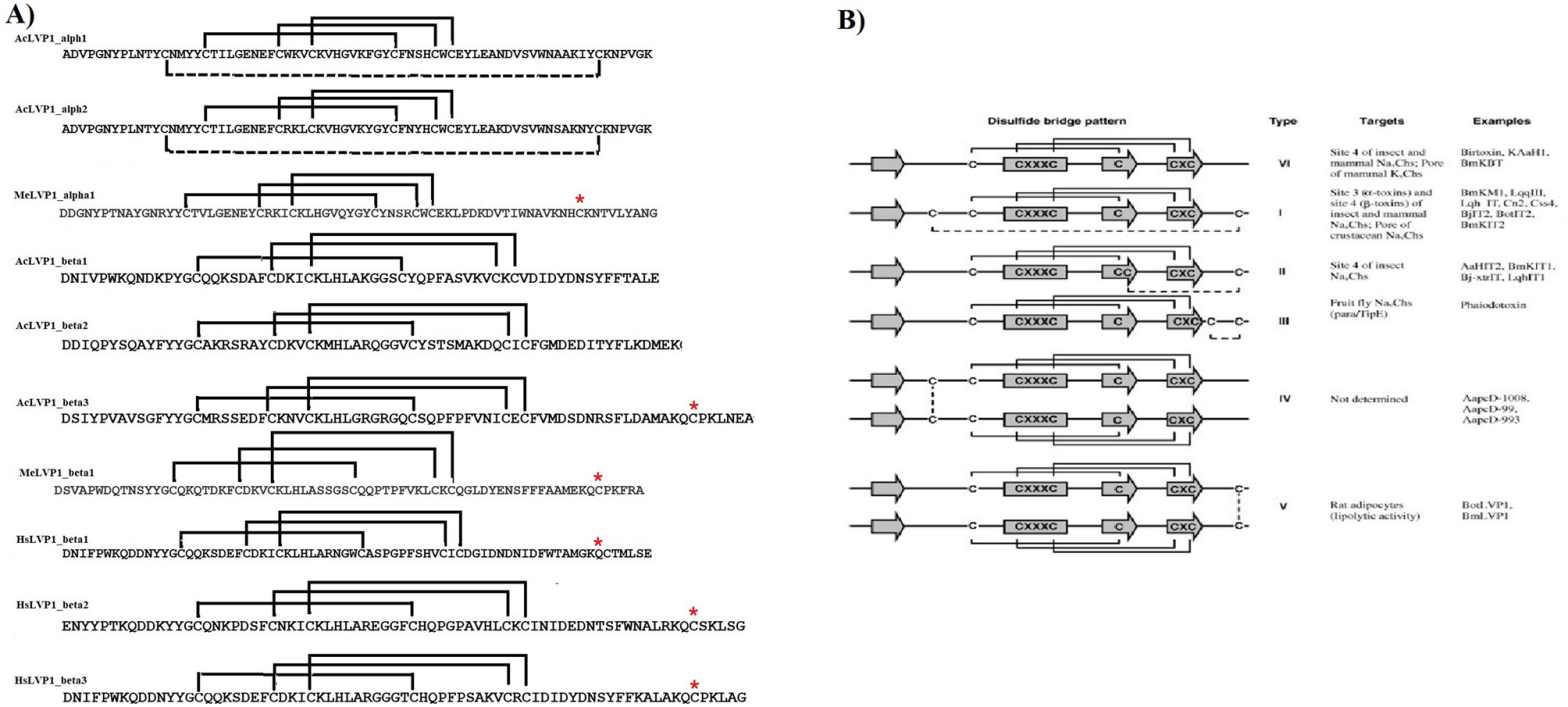
(**A**) Disulfide bridge pattern of identified peptides. The name of each peptide are deposited above the related sequence. The unbounded cysteine residues are shown with red stars. (**B**) Categorization of CSαβ superfamily according to the disulphide bridge pattern scorpion proteins taken from^17^.

Members of the CSαβ superfamily consist of six or eight cysteine residues. According to the number of disulfide bridges and the location of the fourth one, the CSαβ superfamily is categorized into six groups (I-VI) (Fig. 5)^22^. The disulfide bridge pattern of the identified peptides was shown in Fig. 5 to compare with the six identified groups of CSαβ superfamily proteins.

### Phylogenetic relationships based on LVP1

**Figure 6:**
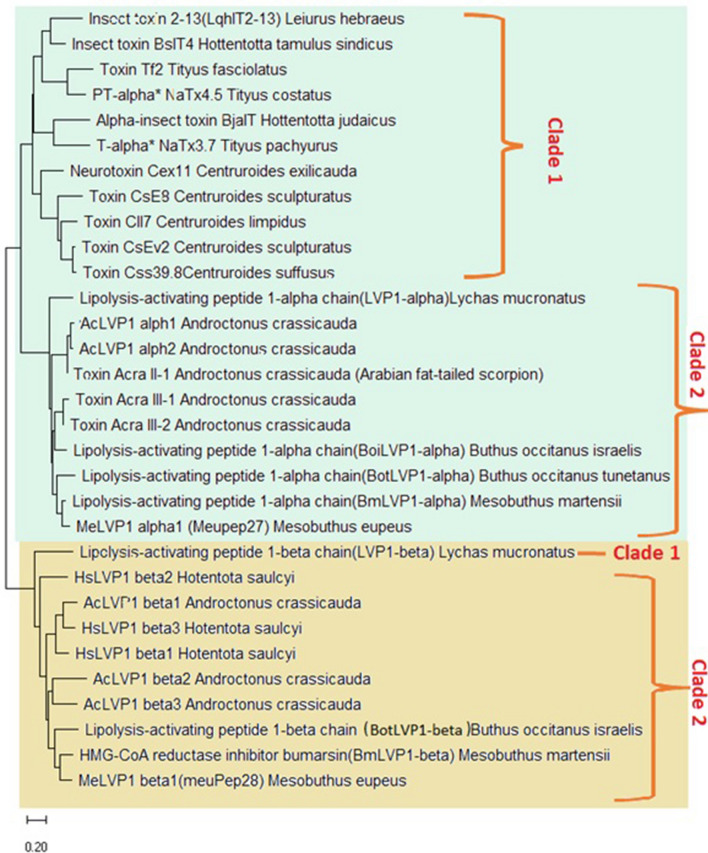
Phylogenetic tree inferred from amino acid sequences of scorpion LVP1-alpha and LVP1-beta. Results from the neighbour-joining bootstrap analyses were mapped. Monophyletic groups A and B are shown is light green and light brown, respectively.

The phylogenetic relationships according to LVP1-alphas and betas are shown in Fig. 6. The phylogenetic tree is divided into two main monophyletic groups, A and B. The proteins in group A (light green) are all LVP1-alphas, and the members of group B (light brown) are all LVP1-betas. Group A is subdivided into two clades. Clade 1 is composed of LVP1-alphas related to species of *Centruroides*, *Leiurus*, and *Tityus* genus, and clade 2 encompass LVP1-alphas of *Lychas mucronatus*, *A. crassicauda* (here identified proteins plus previously identified proteins), *Buthus occitanus*, *M. martensii* and here identified MeLVP1_alpha1 from *M. eupeus*. None of the identified LVP1-alphas belong to clade 1. In clade 2, two isoforms of LVP1-alphas from *A. crassicauda* (AcLVP1_alpha1 and AcLVP1_alpha2) clustered as s sister group which were diverged from an ancestor from which toxin Acra II-1 (from Arabian A. *crassicauda*) was deviated. The other here identified LVP1-alpha, MeLVP1_alpha1 (or meuPep27) from *M. eupeus* formed a sister group with BmLVP1-alpha from *M. martensii*. This could be because *M. eupeus* and *M. martensii* both belong to the same genus. This group has a common ancestor with BotLVP1-alpha from *B. occitanus.* Since *B. occitanus* is a species of the Buthideae family like *M. eupeus* and *M. martensii*, such a position in the phylogenetic tree is justified. Although A. *crassicauda* and *M. eupeus* are also in the same family, MeLVP1_alpha1 and AcLVP1_alpha1,2 proteins are more distant in clade 2. AcLVP1_alpha1,2 contain eight cysteine residues and four disulfide bridges, while MeLVP1_alpha1 contains seven cysteine residues. A possible scenario is that these proteins diverged from a common ancestor. However, during evolution, eight cysteine residues have changed to another amino acid, and a free cysteine has constructed to form a dimer protein.

Monophyletic group B is also subdivided into two main clades. Clade 1 is LVP1-beta from *L. mucronatus*, and clade two is subdivided further into two branches. One includes HsLVP1_beta2, and the other includes the ancestor of LVP1-betas of *B. occitanus*, *M. martensii*, and all the other LVP1-betas identified here. It means HsLVP1_beta2 diverged from the same ancestral protein that gave rise to BmLVP1-beta, BotLVP1-beta, AcLVP1_beta1,2,3, HsLVP1_beta1,2,3, and MeLVP1_beta. It is noteworthy that HsLVP1_beta3 and AcLVP1_beta1, forming a sister group, are diverged from a common ancestor along with HsLVP1_beta1. It means the HsLVP1_beta3 isoform deviated from a common ancestor with AcLVP1_beta1 and is at a further evolutionary distance than the HsLVP1_beta1 isoform.

Like the MeLVP1_alpha1, MeLVP1_beta1 (or meuPep28) clustered in a sister group with a protein from *M. martensii*, BmLVP1-beta. This group diverged from the same ancestor that gave rise to BotLVP1-beta from *B. occitanus.* AcLVP1_beta2 and AcLVP1_beta3 are also clustered in a sister group, whose ancestor together with the ancestor of MeLVP1_beta1, BmLVP1-beta, and BotLVP1-beta have diverged from the same ancestor.

## Discussion

Scorpion venom is a rich source of biologically active peptides and proteins that some of which have pharmaceutical properties. So, the identification of venom molecules can be extremely beneficial. In the current study, we aimed to find different isoforms of subunits of a medicinal potent scorpion protein, LVP1, in four medically important scorpion species, *H. saulcyi*, *M. eupeus*, *A. crassicauda*, and *H. lepturus*, using the RNA-sequencing approach. Analysis of the obtained transcriptome revealed that there are some isoforms of alpha and beta subunits of LVP1 in the venom gland of *A. crassicauda* and *M. eupeus*. In contrast, only the beta isoform is expressed in the venom gland of *H. saulcyi*, and none of the subunits of LVP1 were found in the venom gland of *H. lepturus*.

*H. saulcyi*, *M. eupeus*, and *A. crassicauda* species belong to the Buthidea family, while *H. lepturus* is a member of the Hemiscorpiidae family^34^. So the absence of LVP1 in the venom gland of scorpion *H. lepturus* may be attributed to interspecifically variation in gene expression and venom components, related to differences in evolutionary ancestry of *H. lepturus* compared to the other scorpion species examined in this study. However, it must be pointed out that such variation in the venomous animals also occur intraspecifically^35^. Some studies considered these differences to be due to natural selection for the optimization of venom to diet^36,37^. Even low-expression toxins with significant consequences have been found^38^. Some other studies have explored the effect of climate variation^39^ and geographical differences in intraspecific variation in venom composition^1^.

All identified proteins here presented 50% or higher sequence identity with the referral proteins (BmLVP1-alpha, BotLVP1-alpha, BmLVP1-beta, BotLVP1-beta). It is believed that when two proteins share sequence identity, it indicates similar structure and function. Lower levels of sequence similarity between protein sequences may indicate some relationship between their structures and functions^40^. The threshold of sequence similarity sufficient for structural homology depends strongly on the length of the alignment. As a rule of thumb, for protein sequences longer than 80 amino acids, > 24.8% sequence identity shows similar structure and function^41^. However, some structure alignments have interestingly identified homologous protein pairs with less than 10% sequence identity^42^. The length of all identified proteins here is more than 82 amino acids. So, the calculated sequence identity (> 50%) can verify the similarity of the structure and function of referral proteins with the corresponding identified proteins.

Among the identified proteins, MeLVP1_alpha1 and MeLVP1_beta1 have the highest sequence similarity with BmLVP1 and BotLVP1, i.e., MeLVP1_alpha 88.66% similar to BmLVP1-alpha and MeLVP1_beta1 75.53% similar to BmLVP1_beta. However, Intron–exon pattern analysis of LVP1 subunit genes led to the perdition of two exons and introns with equal size in all identified proteins.

In addition to the fact that the alignment of the proteins showed a significant similarity between BmLVP1-alpha, BotLVP1-alpha, AcLVP1_alph1, AcLVP1_alph2, and MeLVP1_alpha1, the phylogenic analysis has also determined a common ancestor for them. The same story happened for LVP1_ beta isoforms, i.e., BmLVP1-beta and BotLVP1-beta have significant amino acids identity and common ancestor with AcLVP1_beta1, AcLVP1_beta2, AcLVP1_beta3, MeLVP1_beta1, HsLVP1_beta1, HsLVP1_beta2, and HsLVP1_beta3.

The molecular weight, ranging from 7288 g/mol to 9894 g/mol, was calculated for the alpha and beta subunits of the identified LVP1s. In the previous studies, the molecular weight of 8877 g/mol was obtained for the alpha subunit of BotLVP1, and 8807 g/mol and 8132 g/mol were determined for the beta subunits of BotLVP1 and BmLVP1, respectively^16,19^.

Scorpion venom peptides have revealed different functions, but a common, typical folding, CSαβ, has been found in the structure of some of them. These venom peptides were structurally categorized in a group as CSαβ superfamily^43,44^. All here identified isoforms of LVP1-alpha and LVP1-beta contain also determinants of CSαβ folding. However, they have differences in the numbers of the strands of the beta-sheet. AcLVP1_alpha1 and MeLVP1_alpha1 consist of an antiparallel double-stranded beta-sheet, while the others contain a triple-stranded beta-sheet.

Members of the CSαβ superfamily contain six or eight cysteine residues, which, by forming disulfide bridges, stabilize the corresponding protein structure. Three disulfide bridges are a conserved feature in this family, but the position of the fourth disulfide bridge varies among the different members of this family. Considering the number of disulfide bridges and the location of the fourth one, CSαβ proteins are categorized into six groups (Fig. 5)^22^. AcLVP1_alpah1, 2 with eight cysteines are categorized in group I, while AcLVP1_beta1, 2 with six cysteine residues are classified in group VI. MeLVP1_apha1, MeLVP1_beta1, AcLVP1_beta3, and HsLVP1_beta1, 2, 3 all encompassed seven cysteine residues and are assorted in group V. From these three groups, just group V has the potential of forming the dimer because of having an extra cysteine, which is not in bond with any other cysteine. So, MeLVP1_apha1, MeLVP1_beta1, HsLVP1_beta1, 2, 3 can potentially be a dimer.

As described above, LVP1 exists in two forms: heterodimer (containing alpha and beta subunits) and homodimer (containing two beta subunits). LVP1 in the heterodimer form was a simulator of lipolysis in mouse adipocytes^20,21^, and the homodimer form of LVP1-beta inhibited the activity of HMG-CoA reductase^16^. Both BotLVP1 and BmLVP1, along with MeLVP1_apha1, MeLVP1_beta1, AcLVP1_beta3, and HsLVP1_beta1, 2, 3 took in the V category, in which six of cysteine residues involved in the formation of three intermolecular disulfide bridges and the extra cysteine take into an intramolecular disulfide bridge to form a dimer molecule, either a homodimer or a heterodimer^16,19,22^. So, MeLVP1_apha1, MeLVP1_beta1, AcLVP1_beta3, and HsLVP1_beta1, 2, 3 can potentially be a dimer.

There is some evidence that RNA editing is responsible for altering the fourth cysteine residue. RNA editing involves modifying transcripts or inserting and deleting specific sequences to produce alternative protein products with different functions^45^. Similarly, Zhu and Gao interpreted the functional switch of BmLVP1 from adipocyte lipolysis to neurotoxicity by altering the disulfide bridge pattern of the peptides^17^. It seems such a process is responsible for the different patterns seen in the structure of the identified peptides.

## Conclusion

Some studies have identified LVP1s from the scorpion venom as a lipolysis inducer and suggested them be used as new candidates for hyperlipidemia treatment. Due to the pharmacological potential of LVP1, this study provided relevant findings to some new isoforms related to subunits of this protein from three scorpion species. Here, we reported the information related to the transcriptome of *H. saulcyi*, *A. crassicauda*, and *M. eupeus*, with a focus on the identification and characterization of gene and protein of LVP1, which can provide insight into the identification of the similar proteins in the insects. According to similarity, genes encoding isoforms of LVP1s in scorpions are classified into two groups: alpha and beta. We found both subunits in the examined scorpion species, except for *H. lepturus*. The structure of all identified proteins was determined. Understanding the structures of proteins could give us hints about the family of a protein and how a protein works, which can allow us to create hypotheses about how to use them in pharmacological fields or modify them to make more beneficial proteins. The difference in the number of cysteine residues in the identified proteins has caused these proteins to be placed in different groups with different tonalities in dimer formation. AcLVP1_alph1, AcLVP1_alph2, AcLVP1_beta1, and AcLVP1_beta2 cannot form a dimer, while MeLVP1_alpha1, AcLVP1_beta3, HsLVP1_beta1, HsLVP1_beta2, HsLVP1_beta3, and MeLVP1_beta1 can form a dimer through a free cysteine residue. In addition, variability in amino acid composition in the identified proteins and similar proteins in other species, can also lead to changes in the structure and eventually changes in the function (ability and adaptability) of the proteins. Taken together, physico-chemical characterization and protein structure determination of LVP1s, along with phylogenetic analysis, may lay the foundation and shed light on assessing the potential of the scorpion-derived LVP1s for developing novel functional medicine against hyperlipidemia.

## Data Availability

The mRNA and protein sequences reported in this paper are available in the NCBI under the accession numbers OP609899, OP609900, OP609901, OP609902, OP609903, OP612332, OP612333, OP612334, KU513844, and KU513847.
